# Primary Extracranial Meningiomas of the Head and Neck

**DOI:** 10.3390/life11090942

**Published:** 2021-09-09

**Authors:** Giuseppe Emmanuele Umana, Gianluca Scalia, Atul Vats, Gianluca Pompili, Fabio Barone, Maurizio Passanisi, Francesca Graziano, Rosario Maugeri, Maria Grazia Tranchina, Sebastiano Cosentino, Massimo Ippolito, Santino Ottavio Tomasi, Giuseppe Raudino, Bipin Chaurasia, Domenico Gerardo Iacopino, Giovanni Federico Nicoletti, Salvatore Cicero, Lidia Strigari, Rosario Emanuele Perrotta

**Affiliations:** 1 Trauma Center, Gamma Knife Center, Department of Neurosurgery, Cannizzaro Hospital, 95125 Catania, Italy; fbarone1969@gmail.com (F.B.); mpassanisi@tiscali.it (M.P.); cicerosalvatore@yahoo.it (S.C.); 2 Department of Neurosurgery, Highly Specialized Hospital and of National Importance “Garibaldi”, 95125 Catania, Italy; gianluca.scalia@outlook.it (G.S.); fragraziano9@gmail.com (F.G.); gfnicoletti@alice.it (G.F.N.); 3 Neurosurgery Department, James Cook University Hospital, Middlesbrough TS1, UK; vatsatul7@gmail.com; 4 Department of General Surgery and Medical and Surgery Specialities, Section of Plastic Surgery, University of Catania—“Cannizzaro” Hospital, 95125 Catania, Italy; gianluca.pompili@yahoo.it (G.P.); r.perrotta@unict.it (R.E.P.); 5 Neurosurgical Clinic, AOUP “Paolo Giaccone”, Postgraduate Residency Program in Neurological Surgery, Department of Experimental Biomedicine and Clinical Neurosciences, School of Medicine, 90128 Palermo, Italy; rosario.maugeri1977@gmail.com (R.M.); gerardo.iacopino@unipa.it (D.G.I.); 6 Department of Pathological Anatomy, Cannizzaro Hospital, 95125 Catania, Italy; mariagrazia.tranchina@aoec.it; 7 Trauma Center, Gamma Knife Center, Nuclear Medicine Unit, Department of Advanced Technologies, Cannizzaro Hospital, 95125 Catania, Italy; ianocose@hotmail.com (S.C.); ippolitomas@yahoo.it (M.I.); 8 Department of Neurological Surgery, Christian Doppler Klinik Paracelsus Medical University, 5020 Salzburg, Austria; s.tomasi@salk.at; 9 Laboratory for Microsurgical Neuroanatomy, Christian Doppler Klinik, 5020 Salzburg, Austria; 10 Center Humanitas ICC, Misterbianco, Ortho-Neuro, 95125 Catania, Italy; giuraudino@hotmail.it; 11 Department of Neurosurgery, Neurosurgery Clinic, Birgunj 44300, Nepal; trozexa@gmail.com; 12 Department of Medical Physics, IRCCS Azienda Ospedaliero-Universitaria di Bologna, 40122 Bologna, Italy; lidia.strigari@aosp.bo.it

**Keywords:** extracranial meningioma, craniotomy, plastic surgery, scalp flap reconstruction

## Abstract

Meningiomas represent the most common benign histological tumor of the central nervous system. Usually, meningiomas are intracranial, showing a typical dural tail sign on brain MRI with Gadolinium, but occasionally they can infiltrate the skull or be sited extracranially. We present a systematic review of the literature on extracranial meningiomas of the head and neck, along with an emblematic case of primary extracranial meningioma (PEM), which provides further insights into PEM management. A literature search according to the PRISMA statement was conducted from 1979 to June 2021 using PubMed, Web of Science, Google Scholar, and Scopus databases, searching for relevant Mesh terms (primary extracranial meningioma) AND (head OR neck). Data for all patients were recorded when available, including age, sex, localization, histological grading, treatment, possible recurrence, and outcome. A total of 83 published studies were identified through PubMed, Google Scholar, and Scopus databases, together with additional references list searches from 1979 to date. A total of 49 papers were excluded, and 34 manuscripts were considered for this systematic review, including 213 patients. We also reported a case of a 45-year-old male with an extracranial neck psammomatous meningioma with sizes of 4 cm × 3 cm × 2 cm. Furthermore, whole-body ^68^Ga-DOTATOC PET/CT was performed, excluding tumor spread to other areas. Surgical resection of the tumor was accomplished, as well as skin flap reconstruction, obtaining radical removal and satisfying wound healing. PEMs could suggest an infiltrative and aggressive behavior, which has never found a histopathological correlation with a malignancy (low Ki-67, <5%). Whole-body ^68^Ga-DOTATOC PET/CT should be considered in the patient’s global assessment. Surgical removal is a resolutive treatment, and the examination of frozen sections can confirm the benignity of the lesion, reducing the extension of the removal of healthy tissue surrounding the tumor.

## 1. Introduction

Meningiomas represent the most common benign histological tumor of the central nervous system; they originate from cellular elements of the meninges, arachnoid cap cells, arachnoid granulations, subarachnoid blood vessels, fibroblasts, and pia mater [1]. Usually, meningiomas are intracranial, showing a typical “dural tail” sign on brain MRI with Gadolinium, but occasionally they can infiltrate the skull or be sited extracranially [2]. Chronic inflammation processes or oral surgery can trigger the proliferation of these ectopic cells or the stimulation of multipotent mesenchymal cells, thus forming extracranial meningiomas (EMs) [3]. Due to their rarity, EMs can be misdiagnosed: their current occurrence is underestimated, affecting their clinical management negatively. Differential diagnoses should be made with schwannomas, soft tissue perineuriomas, neurofibromas, and paragangliomas [4,5]. Histologically, perineuriomas share many features with meningiomas, but the latter show higher morbidity and greater incidence of neighboring structures infiltration. Indeed, the management of meningiomas includes radiosurgery to treat eventual tumor remnants, and the surgical strategy is more aggressive if compared to perineuriomas [4,5]. Ultrastructural investigation along with immunohistochemistry study is needed to make a proper meningioma diagnosis [4]. We performed a systematic literature review, and we present an emblematic case of primary neck extracranial meningioma, providing further insights into the behavior of these rare tumors, thus improving their clinical management.

## 2. Materials and Methods

### 2.1. Study Selection

**Figure 1:**
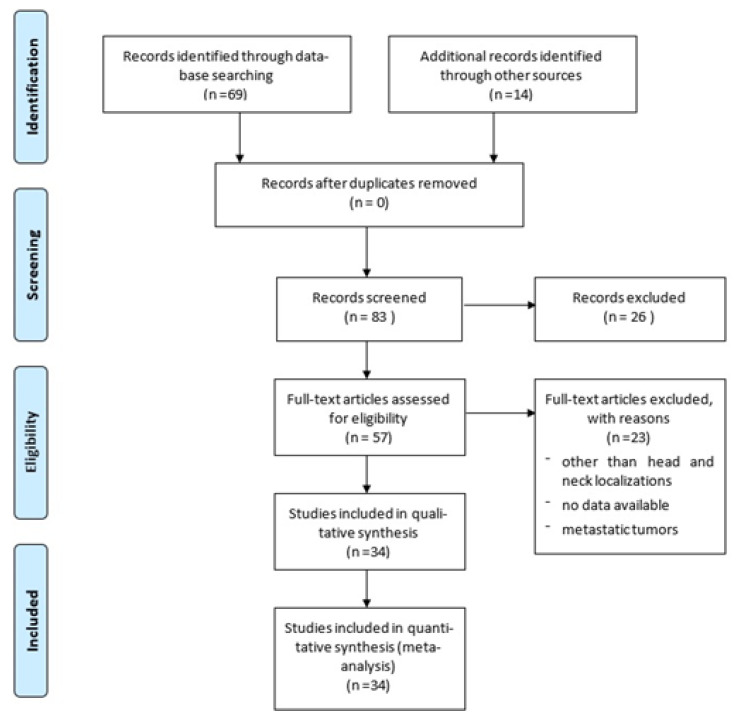
PRISMA flow diagram summarizing our searches and selection of studies included in the literature review of extracranial meningiomas of the head and neck.

A literature search was conducted from 1979 to June 2021 (last published studies in 2020) using PubMed, Web of Science, Google Scholar, and Scopus databases, searching for the following Mesh terms: (primary extracranial meningioma) AND (head OR neck). Papers not written in English were excluded from this study. The PRISMA flow diagram for our search is outlined in Figure 1. The reference lists of all articles discovered in these searches were examined for any additional relevant papers (manual search). Meningiomas involving the spine or extra-neural sites except for head and neck, as well as metastatic locations and malignant histotypes, were excluded. Two reviewers independently screened the titles and abstracts of all extracted citations and then reviewed the full texts of the studies that met the inclusion criteria. A third reviewer settled disagreements.

### 2.2. Data Extraction

One reviewer extracted data from the included articles, then confirmed independently by two additional reviewers. Missing data were either not reported in the original paper or could not be differentiated from other data. All included studies were meticulously reviewed and scrutinized for their study design, methodology, patient characteristics, and extraneural location. Data for all patients were recorded when available, including age, sex, localization, histological grading, treatment, possible recurrence, and outcome. The risk of bias was independently assessed by two reviewers using the Joanna Briggs Institute checklists for case reports and case series (Supplementary Table S1).

## 3. Results

**Figure 2:**
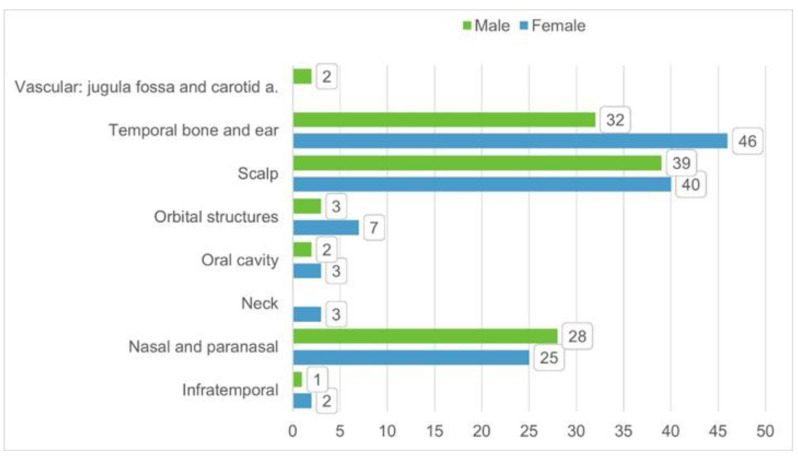
Distribution of patient per sex and tumor/disease localization based on the 34 identified studies from literature.

A total of 83 published studies were identified through PubMed, Web of Science, Google Scholar, and Scopus databases and additional reference list searches from 1979 to date. After a detailed examination of these studies, 49 papers were excluded from our review because they reported localizations other than head and neck or cases of metastatic tumors, or they had no data available. Patients’ demographics are reported in Table 1 [6,7,8,9,10,11,12,13,14,15,16,17,18,19,20,21,22,23,24,25,26,27,28,29,30,31,32,33,34,35,36,37,38,39]. The 34 published studies of this literature review include a total of 231 patients, 106 males (46%) and 124 females (54%), 1 patient not specified (0.4%), with a male/female ratio of 85% (106/124). The reported median age was 36 years (range 0.3–88). The patient distribution by sex and localization is reported in Figure 2.

Surgical resection was performed in 99.1% of patients (229/231). In the remaining four cases, two were partial resections (one patient with neck and the other with oral cavity disease) and one excisional biopsy (patient with oral cavity disease), while the localization and the type of resection were not reported in one patient with neck disease.

**Figure 3:**
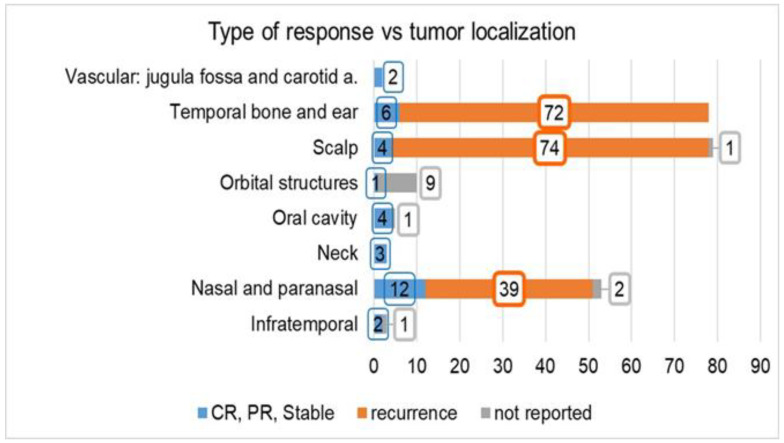
Type of response (complete, partial, stable, or recurrence) reported in the investigated studies vs. tumor localization. The number of cases for which the tumor response is “not reported” is also indicated. Abbreviations: CR: complete response, PR: partial response.

The distribution by type of response versus tumor localization is reported in Figure 3. A higher number of recurrences was observed in lesions located in the scalp (95% = 74/78 patients) and temporal bone and ear (92% = 72/78 patients), while in the lesions located in the nasal and paranasal areas, the rate of recurrence was 76% (39/51 patients). In the remaining localizations, the overall number of patients with a complete response was 12, while the patient outcome was not reported in 11 other patients.

### Case Illustration

**Figure 4:**
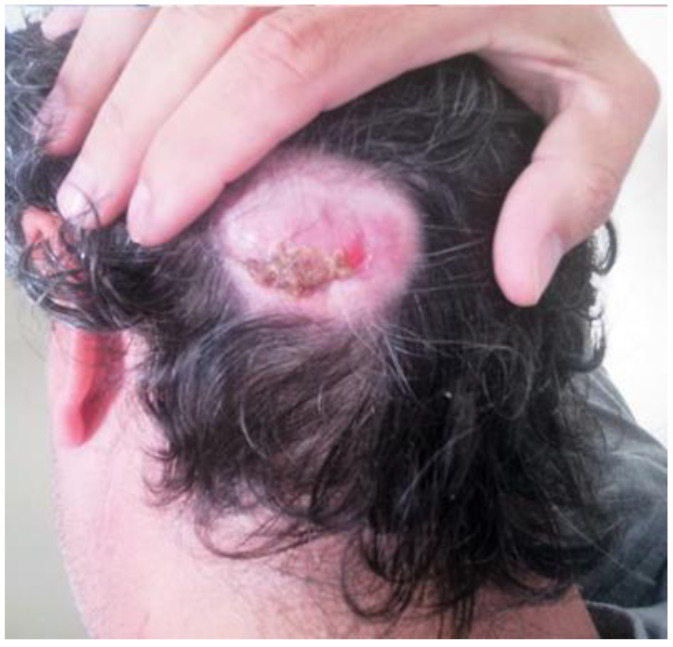
Macroscopic direct posterior view showing a cutaneous nuchal lesion, present from birth, characterized by a callous appearance and superficial protuberance, with a diameter of 4 cm × 3 cm and a thickness of 2 cm.

A 45-year-old male was referred to our department complaining of a cutaneous posterior neck lesion, present since birth, characterized by a callous appearance and superficial protuberance, with a diameter of 4 cm × 3 cm and a thickness of 2 cm (Figure 4). Since the lesion was asymptomatic, the patient had decided not to perform any medical investigations in youth. The lesion did not show any modification over time, but it presented a superficial infection, managed with medication. Then, he decided to perform a radiological imaging study.

**Figure 5:**
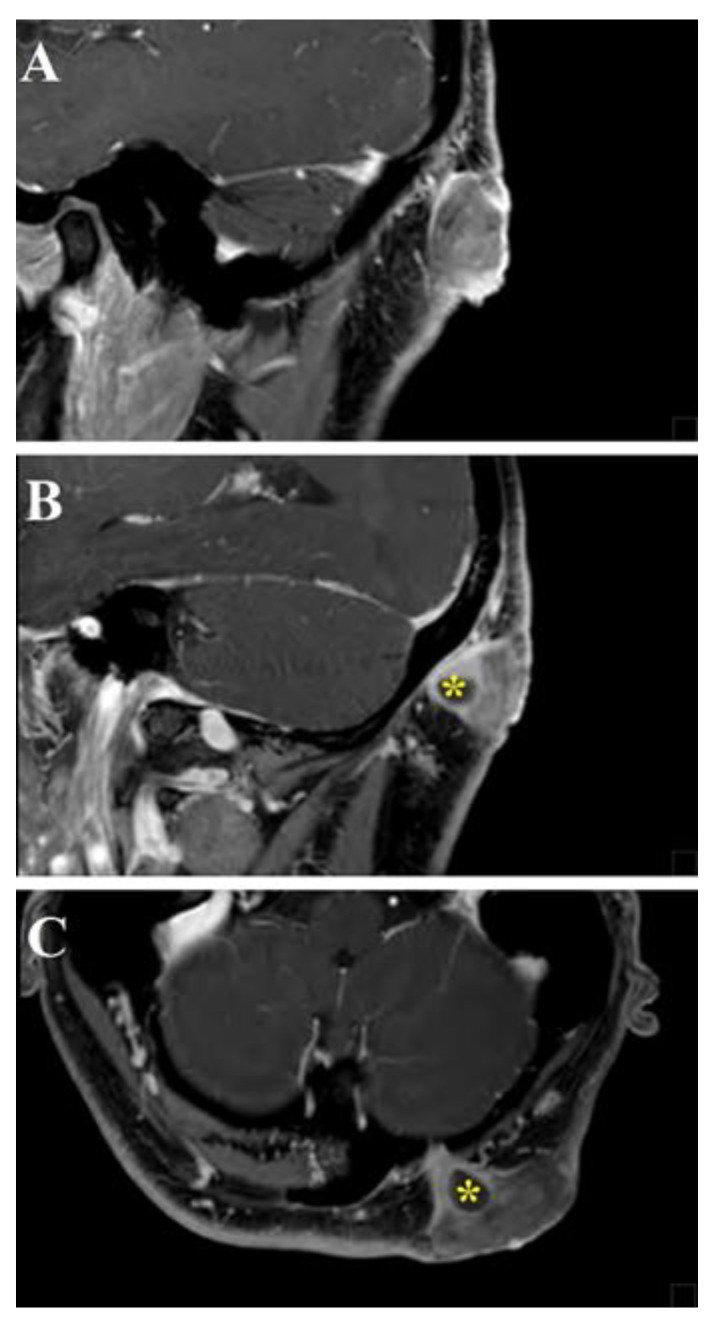
Head and neck MRI sagittal (**A**,**B**) and axial (**C**) images showed a cutaneous mass characterized by homogeneous post-gadolinium enhancement, isointense in T1-weighted sequences, with a characteristic sinus-like propagation that connected the tumor with the cerebellar dura mater through a small passage in the left occipital bone (yellow asterisks).

**Figure 6:**
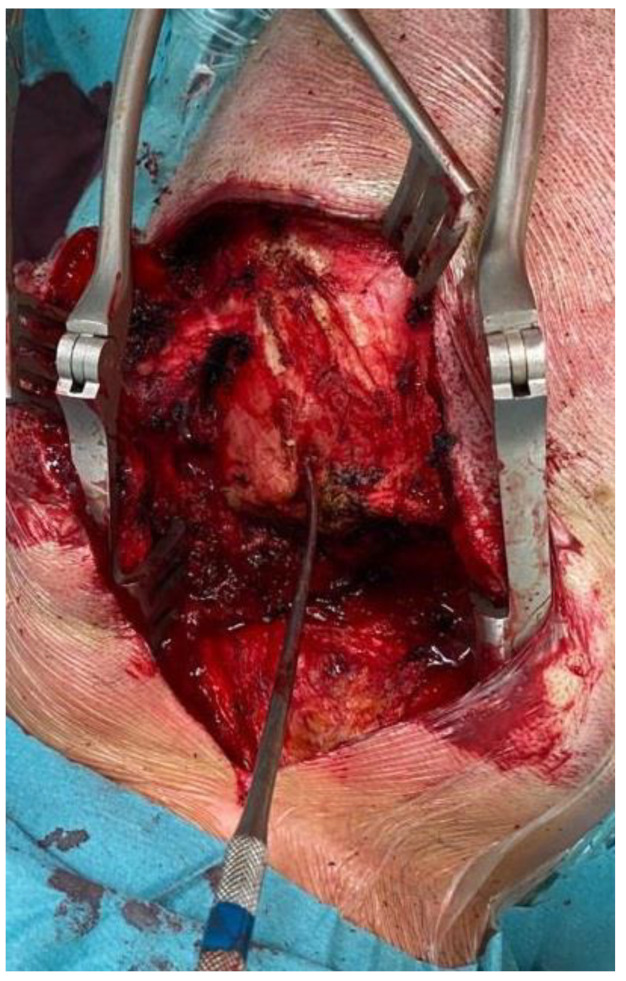
Intraoperative image showing occipital bone exposure and the fistulous tract with a Penfield dissector as a landmark.

**Figure 7:**
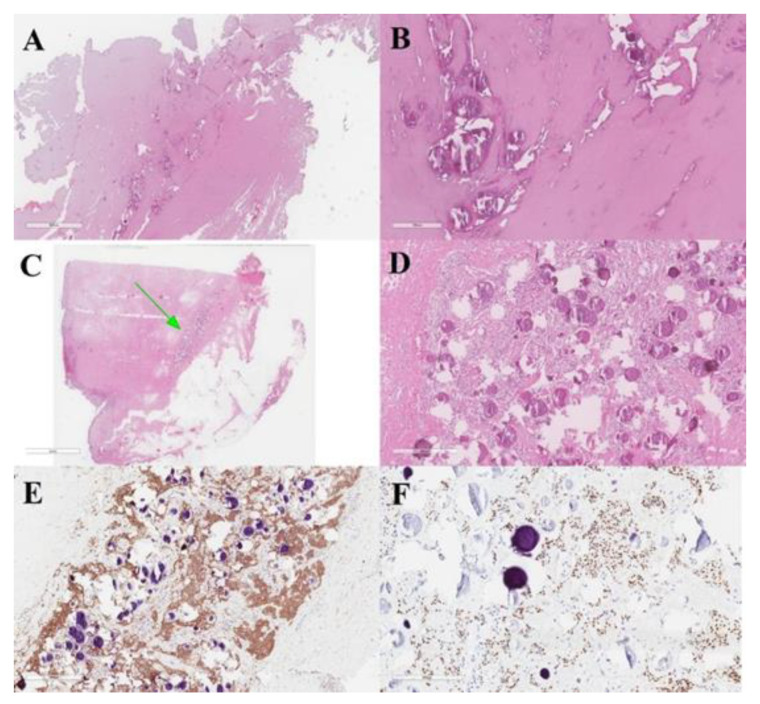
Meningeal fistulous tract of the cervical cutis, represented by fibrous tissue infiltrated by meningioma with psammomatous bodies (**A**,**B**). Dermo-hypodermic localization of predominantly fibrous meningioma with WHO grade I psammomatous bodies (green arrow) (**C**,**D**). Immunostaining was positive for EMA (**E**) and progesterone (**F**).

**Figure 8:**
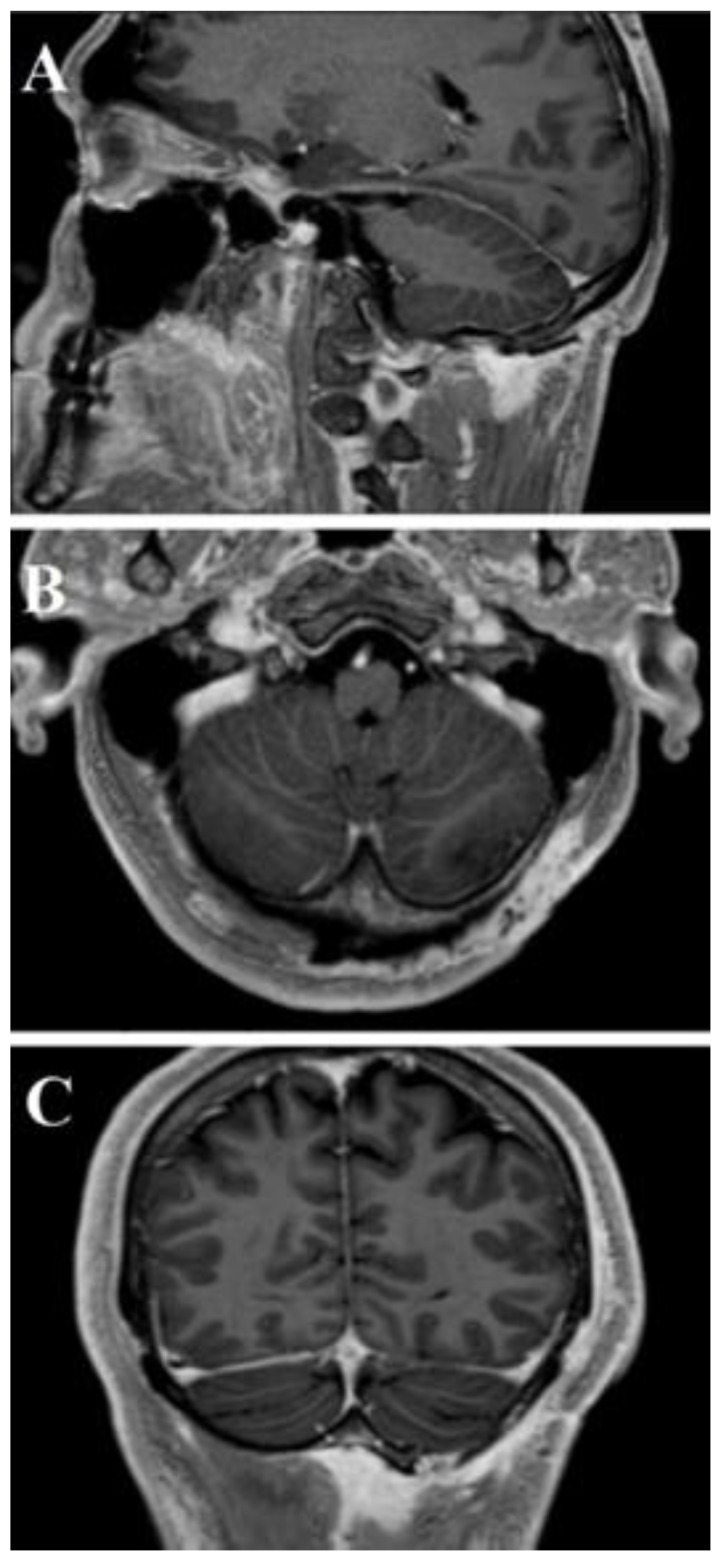
Post-operative head and neck MRI T1-WI with Gadolinium—sagittal (**A**), axial (**B**), and coronal (**C**) areas showed absence of residual tumor.

The patient underwent a head and neck MRI with Gadolinium, which showed a cutaneous mass characterized by homogeneous post-contrast enhancement, isointense in T1-weighted sequences, with a characteristic sinus-like propagation that connected the tumor with the cerebellar dura mater through a small defect in the left occipital bone (Figure 5). A whole-body ^68^Ga-DOTATOC PET/CT was performed, excluding a tumor spread in other body areas. After a multidisciplinary meeting, we decided to perform tumor removal and scalp reconstruction. The first stage of the procedure was conducted by neurosurgeons, performing a hockey stick skin incision exposing the occipital bone and the fistulous tract exposure (Figure 6). Small drilling of the bone was performed, and the dural infiltration was removed. Duroplasty was performed with a heterologous patch, Tachosil, and Tissel. The tumor was then removed en bloc and sent for histological examination. The plastic surgeons performed the second stage of the surgery–closure (in layers after careful hemostasis) and skin reconstruction. An occipital rotation flap with a right lateral pedicle was performed to cover the skin defect; a drain was placed, and the scalp was closed. The final pathological examination showed fibrous tissue from meningioma with psammomatous bodies. Figure 7 shows dermo-hypodermic localization of predominantly fibrous meningioma with WHO grade I psammomatous bodies and ulceration of the epidermis. The wound healed well, and the 6-month post-operative MRI scan showed the absence of residual tumor (Figure 8).

## 4. Discussion

PEMs of the head and neck are uncommon, representing 1–2% of all meningioma localizations. They show benign behavior with good outcomes. In the literature, it is possible to find case reports of extracranial meningiomas at the level of the nasal and paranasal cavities [39], and with progressively decreasing frequency at the level of cranial bones [40], middle ear [15], or within the soft tissues of the head and neck. The pathophysiology of PEMs has been associated with defects of cell migration from the neural crest. However, several possible mechanisms have been proposed: origin from arachnoid cells of nerve sheaths protruding from the skull foramina, ectopic arachnoid granulations, related to traumatism, intracranial hypertension (which could cause the movement of groups of arachnoid cells), or, finally, a possible origin from undifferentiated mesenchymal cells [41].

The case presented showed the unique feature of a connection of the tumor, sited at the level of the skin, to the cerebellar dura mater through a small bone defect. We supposed that the tumor could have originated from arachnoid cells at the level of the cerebellar dura mater; then, because of the progressive growth of the tumor, the tumor may have found a weakness in an emissary vein passing along the occipital bone, through which it expanded to the skin layers. According to this theory, our case should be classified as type A of the Hoye classification (extracranial extension of a meningioma with an intracranial origin, secondary) [42].

Based on their histological and immunohistochemical characteristics, PEMs can be classified as common intracranial meningiomas [43]. Epithelial membrane antigen and vimentin are usually expressed, protein S-100 may show variability, and acidic gliofibrillary protein is usually negative. The presence of these tumors in extracranial sites could suggest an infiltrative and aggressive behavior, which has never found a histopathological correlation with malignancy. This observation is supported by a low Ki-67, which is usually less than 5%. PEMs must be differentiated from several neoplasms, both benign and malignant: epithelial tumors, melanoma, olfactory neuroblastoma, angiofibroma, paraganglioma, and ossifying fibroma [43]. The histological and immunohistochemical differential diagnosis is uncomplicated, and, in more complex and rare cases, the study of protein S-100, cytokeratin, and HMB-45 may help overcome any doubts. The clinical outcome and prognosis of PEMs are favorable, in accordance with the benign histological findings [44,45,46]. A radical removal represents the gold standard to prevent any possible growth of the residual tumor. Considering the rarity of these tumors, clinical examination and neuroimaging are not pathognomonic, so the final diagnosis requires histological investigation. Surgical removal is resolutive, and the examination of frozen sections can confirm the benignity of the lesion, reducing the extension of the removal of healthy tissue surrounding the tumor.

## 5. Conclusions

PEMs could suggest an infiltrative and aggressive behavior, which has never found a histopathological correlation with malignancy (low Ki-67, <5%). Considering the rarity of these tumors, clinical examination and neuroimaging are not pathognomonic, so the final diagnosis requires histological investigation. Whole-body ^68^Ga-DOTATOC PET/CT should be considered in the global assessment of the pathology. Surgical removal is the treatment of choice, and the examination of frozen sections can confirm the benignity of the lesion, reducing the extension of the removal of healthy tissue surrounding the tumor.

## Figures and Tables

**Table 1:** Summary of 34 studies focused on extracranial primary meningiomas of the head and neck, including authors and year, patients’ demographics, localization, histology and grading, treatment, recurrence, and outcome.

Authors and Year	Patients (n)	Age/Sex	Localization	Histology and Grading	Treatment	Recurrence	Outcome
Michel et al., 1979 [6]	1	64F	Neck at parotid (associated with a synchronous intracranial syncytial meningioma of the frontal lobe)	Fibrous meningioma	Resection of the intracranial tumor only and biopsy of the neck tumor	No recurrence and stability of the neck tumor	Transient right arm weakness
Weinberger et al., 1985 [7]	1	Not available	Nasopharynx	Meningioma	Transpalatal flap	Not available	Not available
Friedman et al., 1990 [8]	5	4M, 1F, 17–53 years	2 infratemporal, 2 parapharyngeal, 1 nasal cavity	1 syncytial, 4 transitional. (1 final diagnosis of nf ii associated with transitional meningioma in the 17-year-old male patient)	2 transmandibular, 2 infratemporal, 1 facial degloving	1 recurrence at less than 1 year, 1 recurrence at 3 years	Complete recovery
Miyamoto et al., 1995 [9]	2	15F, 13M	2 occipital scalp	1 meningothelial, 1 fibroblastic	Surgical resection	No recurrence	Complete recovery
O’Reilly et al., 1998 [10]	1	59M	Mesotympanum	Meningioma	Mastoidectomy	No recurrence at 6 months	Complete recovery
Jabor et al., 2000 [11]	1	7F	Facial nerve in the fallopian canal	Meningioma	Not available	Not available	Not available
Kishore et al., 2000 [12]	1	44F	Soft palate	Hyalinized and vascular meningioma	Excisional biopsy	No recurrence	Complete recovery
Sen and Saha, 2001 [13]	1	8F	Right maxillary antrum, right nasal cavity, nasopharynx	Psammomatous	Surgical resection	No recurrence	Complete recovery
Hameed et al., 2002 [14]	1	22F	Sublingual	Meningioma	Surgical resection	Not reported	Not reported
Thompson et al., 2003 [15]	36	24F, 12M, 10–80 years	Ear and temporal bone	Meningothelial (n = 33), psammomatous (n = 2), and atypical (n = 1)	Surgical resection	10 patients developed a recurrencefrom 5 months to 2 years later	Five patients died with recurrent disease (mean, 3.5 years), and the remaining 30 patients lived (n 25, mean: 19.0 years) or died (n 5, mean: 9.5 years) ofunrelated causes without evidence of disease
Shaw et al., 2004 [16]	1	29M	Occipital scalp	Meningothelial meningioma	Surgical resection	No recurrence at 12 months	Complete recovery
Eshete et al., 2005 [17]	1	70M	Parietal scalp	Meningothelial meningioma	Surgical resection	No recurrence	Not available
Jian et al., 2005 [18]	1	19F	Pterygopalatine fossa	Meningioma	Surgical resection	No recurrence	Not reported
Ouazzani et al., 2007 [19]	1	27F	Parietal scalp	Lymphoplasmacyte-rich meningioma	Surgical resection	No recurrence	Complete recovery
Rushing et al., 2009 [20]	146	74F, 72M; 0.3–88 years	Scalp skin (40.4%), ear and temporal bone (26%), and sinonasal tract (24%).	Meningothelial (77.4%), followed by atypical (7.5%), psammomatous (4.1%), and anaplastic (2.7%)	Surgical resection	Recurrences were noted in 26 (23.6%) patients	Recurrent disease was persistent in 15 patients (mean, 7.7 years): 13 patients died with disease and two lived; the remaining patients were disease-free (alive 60, mean 19.0 years, dead 35, mean 9.6 years)
Rutt et al., 2009 [21]	1	45M	Jugular fossa	Meningioma	Surgical resection	No recurrence at 1 year	Complete recovery
Alzarae et al., 2010 [22]	1	60M	Nasal septum	Meningioma	Surgical resection	No recurrence at 4 years	Complete recovery
George et al., 2010 [23]	1	35F	Middle-ear mass	Meningothelial meningioma	Surgical resection, tympanoplasty	No recurrence at 3 months	Complete recovery
Deshmukh et al., 2011 [24]	1	18F	Right submandibular	Meningothelial meningioma	Surgical resection	No recurrence at 2 years	Complete recovery
Aiyer et al., 2012 [25]	3	13F, 45F, 23F	Paranasal sinuses	Meningioma, meningioma, fibroblastic meningioma	Frontoethmoidectomy, left lateral rhinotomy approach	No recurrence at 1 year, 8 months, 6 months	Complete recovery
Baek et al., 2012 [26]	1	44M	Nasal cavity	Meningioma WHO III	Surgical resection	Recurrence 2 years after surgery, treated with local external radiotherapy (6840 cgy in 38 fractions); after 2 more months, the patient developed malignant chest meningiomas (WHO III), surgically removed, and then developed multiple cutaneous tumors addressed to palliative treatment	Died 3 months after the last surgery
Possanzini et al., 2012 [27]	3	38F, 69F, 34F	Temporal muscle, parapharyngeal, nasal mass	Epithelioid, s-100 positive, negative for gliofibrillary protein, cd31, cytokeratin Pool, cd34, smooth muscle actin, chromogranin a, Synaptophysin and melanocytic antigen. Immunoreactive Ki-67 cells were less than 5%	Surgical resection	Disease-free at 3 years	Complete recovery
Zulkiflee et al., 2012 [28]	1	54M	Bifurcation of the left common carotid artery	Meningioma of the hypoglossal nerve	Transcervical excision	No recurrence at 2 years	Complete recovery
Maeng et al., 2013 [29]	1	66F	Right cheek	Meningothelial meningioma	Lateral rhinotomy	No recurrence at 18 months	Complete recovery
Ocque et al., 2014 [30]	8	26–64/3M–5F	1 ear, 2 orbit,1 neck, 2 sphenoid sinus, 1 sella tuberculum, 1 parapharyngeal	Meningioma	Surgical resection	Not reported	Not reported
Albsoul et al., 2015 [31]	1	55F	Neck	Meningothelial meningioma	Right cervical exploration for excisional biopsy and, because of the encasement of the right internal jugular vein, partial excision of the mass.	Stable after 8-month follow-up	Stable
Asil et al., 2015 [32]	1	64M	Right frontotemporal	Intradiploic meningioma	Surgical resection	No recurrence at 2 months	No deficits
Janakiram et al., 2015 [33]	2	40M, 36M	Nasal cavity, frontal sinus	Meningothelial pattern. Psammoma bodies were seen in between. There was no nuclear atypia or mitotic activity. Immunohistochemistry was positive for epithelial membrane antigen (ema)	Endoscopic endonasal approach	No recurrence at 2 years	Complete recovery
Mondal et al., 2015 [34]	1	2M	Mass in the ethmoid sinus	Meningioma	Resection	No recurrence at 3 months	Complete recovery
Yang et al., 2015 [35]	1	16M	Nasal floor	Meningioma	Surgical resection	No recurrence at 6 months	Complete recovery at 1 week
Ma et al., 2016 [36]	1	18M	Left side of the tongue	Atypical meningioma	Lip-split mandibulotomy approach	No recurrence at 21 months	Progressive recovery and good functional speech and swallowing
Lee et al., 2017 [37]	1	77F	Left eyebrow	Meningothelial meningioma	Trans-eyebrow removal	No recurrence at 10 months	Not reported
Rege et al., 2017 [1]	1	35M	Right retromolar	Meningioma, antibodies against ema, vimentin, and Cd34 were positive	Partial resection of the mandible and reconstruction with autogenous iliac tricortical bone	No recurrence at 5 years	Complete recovery
Radke et al., 2018 [38]	1	54F	Nasolacrimal sac/fossa	Epithelioid meningioma	Dacryocystorhinostomy with exploration	No recurrence at 3 months	Tearing persisted, conjunctivodacryocystorhinostomy was planned after 1 year to ensure the tumor did not recur
Umana et al., 2021	1	45M	Occipital scalp	Psammomatous meningioma	Surgical resection	No recurrence at 6 months	Complete recovery

## Supplementary Materials

The following are available online at https://www.mdpi.com/article/10.3390/life11090942/s1, Figure S1: PRISMA flow diagram summarizing our searches and selection of studies included in the literature review of extracranial meningiomas of the head and neck., Table S1: Summary of 34 studies focused on extracranial primary meningiomas of the head and neck, including authors and year, patients’ demographics, localization, histology and grading, treatment, recurrence, and outcome.
