# Supplementary material for: Primary Extracranial Meningiomas of the Head and Neck
Source: Life (Basel). 2021 Sep 9;11(9):942. doi: 10.3390/life11090942 (PMC8468587; doi:10.3390/life11090942)
Supplement: Supplementary file 1 [file life-11-00942-s001.zip › life-1318769-supplementary.pdf]

Supplementary Materials of Primary Extracranial Meningiomas of the Head and Neck

Table S1. Risk of bias assessments for included studies.

**JBI Checklist for Case Reports–Criteria**

Copyright: © 2021 by the authors. Licensee MDPI, Basel, Switzerland. This article is an open access article distributed under the terms and conditions of the Creative Commons Attribution (CC BY) license (http://creativecommons.org/licenses/by/4.0/).

1. Were patient's demographic characteristics clearly described?
2. Was the patient's history clearly described and presented as a timeline?
3. Was the current clinical condition of the patient on presentation clearly described?
4. Were diagnostic tests or assessment methods and the results clearly described?
5. Was the intervention(s) or treatment procedure(s) clearly described?
6. Was the post-intervention clinical condition clearly described?
7. Were adverse events (harms) or unanticipated events identified and described?
8. Does the case report provide takeaway lessons?
Responses Options: Yes, No, Unclear, Not Applicable (NA)
Quality Rating: Poor 0 – 2; Fair 3 – 5; Good 6 – 8

**Study**	**1**	**2**	**3**	**4**	**5**	**6**	**7**	**8**	**Rating**
Michel et al., 1979 [6]	©	Yes	Yes	Yes	Yes	Yes	Yes	Yes	8 – Good
Weinberger et al., 1985 [7]	Yes	Yes	Yes	Yes	Yes	Yes	Yes	Yes	8 – Good
O'Reilly et al., 1998 [10]	Yes	Yes	Yes	Yes	No	Yes	Yes	Yes	7 – Good
Jabor et al., 2000 [11]	Yes	Yes	Yes	Yes	Yes	Yes	Yes	Yes	8 – Good
Kishore et al., 2000 [12]	Yes	Yes	Yes	Yes	Yes	Yes	Yes	Yes	8 – Good
Sen and Saha, 2001 [13]	Yes	Yes	Yes	Yes	Yes	Yes	Yes	Yes	8 – Good
Hameed et al., 2002 [14]	Yes	Yes	Yes	Yes	Yes	Yes	Yes	Yes	8 – Good
Shaw et al., 2004 [16]	Yes	Yes	Yes	Yes	Yes	Yes	Yes	Yes	8 – Good
Eshete et al., 2005 [17]	Yes	Yes	Yes	Yes	Yes	Yes	Yes	Yes	8 – Good
Jian et al., 2005 [18]	Yes	Yes	Yes	Yes	Yes	Yes	Yes	Yes	8 – Good
Ouazzani et al., 2007 [19]	Yes	Yes	Yes	Yes	Yes	Yes	Yes	Yes	8 – Good
Rutt et al., 2009 [21]	Yes	Yes	Yes	Yes	Yes	Yes	Yes	Yes	8 – Good
Alzarae et al., 2010 [22]	Yes	Yes	Yes	Yes	Yes	Yes	Yes	Yes	8 – Good
George et al., 2010 [23]	Yes	Yes	Yes	Yes	Yes	Yes	Yes	Yes	8 – Good
Deshmukh et al., 2011 [24]	Yes	Yes	Yes	Yes	Yes	Yes	Yes	Yes	8 – Good
Baek et al., 2012 [26]	Yes	Yes	Yes	Yes	Yes	Yes	Yes	Yes	8 – Good
Zulkiflee et al., 2012 [28]	Yes	Yes	Yes	Yes	Yes	Yes	Yes	Yes	8 – Good
Maeng et al., 2013 [29]	Yes	Yes	Yes	Yes	Yes	Yes	Yes	Yes	8 – Good
Albsoul et al., 2015 [31]	Yes	Yes	Yes	Yes	Yes	Yes	Yes	Yes	8 – Good
Asil et al., 2015 [32]	Yes	Yes	Yes	Yes	Yes	Yes	Yes	Yes	8 – Good
Mondal et al., 2015 [34]	Yes	Yes	Yes	Yes	Yes	Yes	Yes	Yes	8 – Good
Yang et al., 2015 [35]	Yes	Yes	Yes	Yes	Yes	Yes	Yes	Yes	8 – Good
Ma et al., 2016 [36]	Yes	Yes	Yes	Yes	Yes	Yes	Yes	Yes	8 – Good
Lee et al., 2017 [37]	Yes	Yes	Yes	Yes	Yes	Yes	Yes	Yes	8 – Good
Rege et al., 2017 [38]	Yes	Yes	Yes	Yes	Yes	Yes	Yes	Yes	8 – Good
Radke et al., 2018 [39]	Yes	Yes	Yes	Yes	Yes	Yes	Yes	Yes	8 – Good

**JBI Checklist for Case Series–Criteria**
1. Were there clear criteria for inclusion in the case series?
2. Was the condition measured in a standard, reliable way for all participants included in the case series?

3. Were valid methods used for identification of the condition for all participants included in the case series?
4. Did the case series have consecutive inclusion of participants?
5. Did the case series have complete inclusion of participants?
6. Was there clear reporting of the demographics of the participants in the study?
7. Was there clear reporting of clinical information of the participants?
8. Were the outcomes or follow up results of cases clearly reported?
9. Was there clear reporting of the presenting site(s)/clinic(s) demographic information?
10. Was statistical analysis appropriate?
Responses Options: Yes, No, Unclear, Not Applicable (NA)
Quality Rating: Poor 0–3; Fair 4–7; Good 8–10

Study	1	2	3	4	5	6	7	8	9	10	Appraisal
Friedman et al., 1990 [8]	Yes	Yes	Yes	Yes	Yes	Yes	Yes	Yes	No	NA	8 – Good
Miyamoto et al., 1995 [9]	Yes	Yes	Yes	Yes	Yes	Yes	Yes	Yes	No	NA	8 – Good
Thompson et al., 2003 [15]	Yes	Yes	Yes	Yes	Yes	Yes	Yes	Yes	No	NA	8 – Good
Rushing et al., 2009 [20]	Yes	Yes	Yes	Yes	Yes	Yes	Yes	Yes	No	NA	8 – Good
Aiyer et al., 2012 [25]	Yes	Yes	Yes	Yes	Yes	Yes	Yes	Yes	No	NA	8 – Good
Possanzini et al., 2012 [27]	Yes	Yes	Yes	Yes	Yes	Yes	Yes	Yes	No	NA	8 – Good
Ocque et al., 2014 [30]	Yes	Yes	Yes	Yes	Yes	Yes	Yes	Yes	No	NA	8 – Good
Janakiram et al., 2015 [33]	Yes	Yes	Yes	Yes	Yes	Yes	Yes	Yes	No	NA	8 – Good
